# Analysis of Chemical Composition and Biological Activities of *Aloe vera* Rind Extract Obtained by Ultrasound‐Assisted Extraction

**DOI:** 10.1002/fsn3.71264

**Published:** 2025-11-28

**Authors:** Zahra Dehghan, Alireza Vasiee, Fahime Lavi Arab, Farideh Tabatabaee Yazdi

**Affiliations:** ^1^ Department of Food Science and Technology, Faculty of Agriculture Ferdowsi University of Mashhad Mashhad Iran; ^2^ Research Institute of Food Science and Technology (RIFST) Mashhad Iran; ^3^ Department of Immunology, Faculty of Medicine Mashhad University of Medical Sciences Mashhad Iran; ^4^ Immunology Research Center Mashhad University of Medical Sciences Mashhad Iran

**Keywords:** additive, *Aloe vera* rind, pharmaceutical industry, phytochemical, total phenolic

## Abstract

Ultrasound‐assisted extraction of Aloe vera rind yielded significantly more extract (ARE) than conventional maceration, and was therefore selected for in‐depth analysis. GC–MS profiling identified thieno\[2,3‐c]furan‐3‐carbonitrile, 2‐amino‐4,6‐dihydro‐4,4,6,6‐tetramethyl as the predominant compound (49.2%). FTIR spectroscopy confirmed functional groups characteristic of phenolic and flavonoid bioactives. Quantification revealed total phenolic content of 16.88 mg gallic acid equivalents (GAE)/g and total flavonoid content of 32.08 mg quercetin equivalents (QE)/g. Antioxidant efficacy was demonstrated via DPPH radical‐scavenging and FRAP assays, indicating strong free‐radical neutralization and reducing power. Antimicrobial activity assessed by disk diffusion and well diffusion methods showed potent inhibition of *Staphylococcus aureus* and *Listeria innocua* , with minimum inhibitory and bactericidal concentrations (MIC/MBC) of 18.75 mg/mL. Pseudomonas aeruginosa was least sensitive (MIC = 75 mg/mL). SEM examination of ARE‐treated S. aureus and *Escherichia coli* revealed pronounced cell‐wall disruption and nucleic acid leakage. Additionally, cytotoxicity assays against cancer cell lines yielded an IC50 of 27.37 mg/mL, demonstrating moderate anticancer potential. Overall, ultrasound extraction proves an efficient method for isolating bioactive compounds from *Aloe vera* rind. The resulting ARE exhibits notable antioxidant, antimicrobial, and anticancer activities, underscoring its promise for development in pharmaceutical formulations and functional food applications.

## Graphical abstract

The Aloe vera rind extract was obtained using ultrasound‐assisted extraction. The antioxidant properties of this extract were assessed through various tests, including the measurement of phenolic and flavonoid content, as well as the DPPH radical inhibition assay. Additionally, the antimicrobial properties of the extract were evaluated using Minimum Inhibitory Concentration (MIC) and Minimum Bactericidal Concentration (MBC) tests against *Escherichia coli* and *Staphylococcus aureus* bacteria.

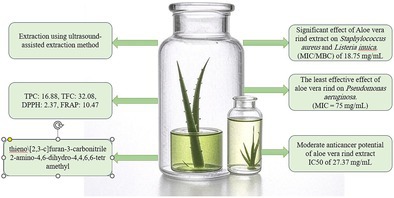

## 1 Introduction

Medicinal and herbal plants have been utilized as therapeutic agents since ancient times, spanning diverse regions worldwide (Behbahani, Yazdi, Shahidi, et al. 2018; Behbahani, Yazdi, Vasiee, and Mortazavi 2018; Ghasemi Pirbalouti et al. 2016). These plants are rich in a diverse array of bioactive compounds, including terpenoids, phenols, glycosides, esters, alcohols, and flavonoids, which play pivotal roles in disease prevention and treatment (Behbahani et al. 2014; Hęś et al. 2019).

*Aloe barbadensis* Miller, commonly known as *Aloe vera* , is a prominent medicinal plant belonging to the Liliaceae family. It is one of the most widely recognized species within the genus Aloe, which comprises approximately 460 species. *Aloe vera* is native to South Africa (Quispe et al. 2018).

Its extensive historical significance has led to its incorporation into traditional medicine practices in cultures such as China, India, and Japan for centuries. Contemporary research has elucidated various medicinal properties associated with *Aloe vera* , including anticancer, antimicrobial, antioxidant, and antidiabetic effects (López et al. 2013; Radha and Laxmipriya 2015).

The leaf segment known as *Aloe vera* rind (AVR) is often regarded as a by‐product. It constitutes approximately 20%–30% of the total leaf weight (Añibarro‐Ortega et al. 2019). The vascular bundles located between the outer green rind and the inner layer of the leaf facilitate the transport of a bitter, yellowish latex. The clear inner pulp, which forms the third layer of the leaf, is predominantly composed of parenchymal tissue cells. This inner gel, commonly referred to as *Aloe vera* gel or fillet, is considered the most valuable component of the plant. It is predominantly utilized in product manufacturing (Guo and Mei 2016).

During the manufacturing process, the inner gel is typically separated from the outer rind. However, the outer rind, which accounts for over 30% of the total leaf weight, generates substantial agricultural waste. Regrettably, this waste often lacks commercial applications and is frequently discarded, composted, or incinerated (Safdar et al. 2017). Although there is limited information available about *Aloe vera* rind compared to the internal gel, several studies have underscored the potential of *Aloe vera* rind as a rich source of bioactive compounds with diverse applications in the food, food packaging, and other sectors. Furthermore, research has investigated the comparative effects of antibiotics and *Aloe vera* on isolated bacteria in the context of skin infections (Arbab et al. 2021), the influence of pomegranate and ARE on *Streptococcus mutans* (Subramaniam et al. 2012), and the antimicrobial activity of zinc oxide nanoparticles synthesized from *Aloe vera* peel extract (Chaudhary et al. 2019).

Plant polyphenols, renowned for their potent antimicrobial and antioxidant properties, have garnered substantial scholarly attention due to their remarkable efficacy in alleviating various diseases associated with oxidative stress, including cancer (Alizadeh Behbahani et al. 2013). Consequently, medicinal plants present promising alternatives to synthetic additives, contributing to the prevention of cancer and microbial foodborne illnesses (Ahmad Nejhad et al. 2023; Behbahani, Yazdi, Shahidi, et al. 2018; Yazdi et al. 2015). Given the persistent challenge of microbial spoilage in food and water, it is imperative to leverage substances that not only possess antimicrobial properties but also pose minimal health risks to consumers. Moreover, the inappropriate and excessive use of antibiotics has led to the emergence of microbial resistance, emphasizing the urgent need to incorporate natural antimicrobials, such as plant‐derived compounds like essential oils and extracts, into societal practices (Behbahani et al. 2019).

The selection of an appropriate extraction method is paramount for optimizing efficiency and achieving high‐quality products (Munekata et al. 2020). Innovative extraction techniques offer several advantages over traditional methods, including reduced organic solvent consumption, preservation of native compounds, and enhanced extraction efficiency. One such technique is ultrasonic‐assisted extraction (UAE), which utilizes high‐frequency sound waves in conjunction with solvents to facilitate the release and diffusion of cellular materials (Yusoff et al. 2022). This method generates acoustic cavitation within the liquid medium, leading to improved mass transfer, which is a significant advantage of UAE. Key advantages of this technique include reduced extraction time, lower energy consumption, and minimal solvent usage compared to conventional extraction methods (Munekata et al. 2020; Yusoff et al. 2022).

Previous research has investigated the extraction of aloesin from *Aloe vera* rind using environmentally friendly solvents such as ethanol, propylene glycol, and glycerol (Añibarro‐Ortega et al. 2019). Additionally, microwave‐assisted extraction has been employed to isolate bioactive compounds from *Aloe vera* peel (Solaberrieta et al. 2022).

The objectives of the present study are to compare the efficacy of maceration and ultrasound extraction methods for extracting extracts from *Aloe vera* rind. Furthermore, we aim to identify the bioactive compounds extracted through the selected method and evaluate the antioxidant, antibacterial, and cytotoxic properties of the *Aloe vera* rind extract (ARE).

## 2 Materials and Methods

### 2.1 Raw Material

The research utilized fresh *Aloe vera* leaves sourced from a nursery in Mashhad, Iran, in accordance with all applicable institutional guidelines and regulations. The plant material was authenticated at the herbarium center of Ferdowsi University of Mashhad, Iran (Voucher sp. No: E‐1597 FUMH). The leaves underwent a meticulous washing process, followed by the careful removal of the outer green rind using a knife. Subsequently, the leaves were dried in an oven at 60°C for a period of 48 h. After drying, the rinds were finely ground into a powder and stored at room temperature for subsequent analyses (Negahdari et al. 2017).

### 2.2 Chemicals

The requisite chemicals for the study, including 2,2‐diphenyl‐1‐picrylhydrazyl (DPPH), 3‐[4 C‐dimethylthiazole‐2‐yl]‐2,5‐diphenyltetrazolium bromide (MTT), quercetin, gallic acid, and Folin–Ciocalteu reagent, were procured from Sigma‐Aldrich, a well‐established supplier based in the United States. These chemicals are specifically designed for utilization within the experimental protocols outlined in the study (Negahdari et al. 2017).

### 2.3 Preparation of Extract

#### 2.3.1 Maceration Extraction

The maceration extraction procedure was performed according to Negahdari et al. (2017), with minor modifications. Specifically, 6 g of *Aloe vera* powder were combined with 120 mL of 80% ethanol. The resulting mixture was agitated for 3 h and then allowed to stand for 24 h. Subsequently, the mixture was filtered through Whatman No. 1 filter paper to obtain a clear liquid. The filtered solution was subsequently dried in an oven at 50°C for 48 h. The weight of the resultant extract was subsequently recorded (Jalil Sarghaleh et al. 2023; Negahdari et al. 2017). It is noteworthy that three separate *Aloe vera* specimens were utilized, and extraction was performed three times for each specimen's rind, resulting in a total of nine repetitions. The average weight obtained from these nine extractions was reported.

#### 2.3.2 Ultrasonic Assisted Extraction (UAE)

The UAE procedure was executed in accordance with the methodology outlined by Faria et al. (2020), with specific modifications. In this method, 6 g of *Aloe vera* powder was combined with 120 mL of 80% ethanol and stirred for 3 h. The mixture was then allowed to rest at room temperature for 24 h. After this incubation, the samples were subjected to sonication using a focused ultrasound device (Misonix Sonicator XL2020 Ultrasonic Liquid Processor, 20 kHz) with a power setting of 40% (220 W) for a duration of 30 min. Following sonication, the samples were centrifuged at 6000 rpm for 10 min, and the resulting supernatants were collected. These supernatants were transferred to a pre‐weighed plate and placed in an oven at 50°C for 48 h to facilitate the drying process. The weight of the extract was subsequently measured after drying (Faria et al. 2020).

#### 2.3.3 Extraction Efficiency

The extraction efficiency (%) was determined according to the method of Rostami and Gharibzahedi (2016) (Rostami and Gharibzahedi 2016) using the following equation:

$$\text{Extraction efficiency}=\frac{W_{\text{final}}-W_{\text{initial}}}{m_{\text{sample}}}\times 100$$

where W_final_ is the combined mass of the collection plate and the recovered extract (g), W_initial_ is the mass of the empty collection plate prior to extraction (g), and m_sample_ is the mass of the original sample subjected to extraction (g).

Prior to each assay, the empty collection plate was weighed to obtain W_initial_. After extraction and transfer of the recovered material onto the plate, the plate plus extract was re‐weighed to yield W_final_. All measurements were performed in triplicate, and the mean extraction efficiency is reported.

### 2.4 Antioxidant Assessment

#### 2.4.1 DPPH Free Radical Scavenging Assay

In this investigation, a prepared stock DPPH solution containing 0.004 g of DPPH dissolved in 100 mL of methanol was transferred into a test tube. Subsequently, 0.1 mL of ARE at a concentration of 1 mg/mL was added to the test tube. The resulting mixture was incubated in the dark for 30 min to allow the reaction to proceed. After the incubation period, the absorbance of the solution was measured at a wavelength of 517 nm. The percentage of DPPH radical scavenging activity was subsequently calculated using the following formula:

$$\text{Scavenging activity}\;\left(\%\right)=\frac{\mathrm{Abs}\;\text{control}-\mathrm{Abs}\;\text{sample}}{\mathrm{Abs}\;\text{control}}\times 100$$

In this equation, “Abs control” refers to the absorbance of the DPPH solution mixed with ethanol, while “Abs sample” denotes the absorbance of the DPPH radical in the presence of the ARE. Additionally, the results were quantified using the IC50 value, which represents the concentration of the antioxidant required to reduce the DPPH concentration to 50% of its initial value. For comparative analysis, butylated hydroxytoluene (BHT) was employed as a positive control in this study (Ghazanfari et al. 2023; Okoh et al. 2014).

#### 2.4.2 Ferric Reducing Antioxidant Potential (FRAP) Assay

The FRAP assay was conducted to evaluate the antioxidant capacity of the ARE. To execute the assay, the ARE was prepared at a concentration of 1 mg/mL by dissolving it in ethanol. Subsequently, 0.2 mL of the extract solution was combined with 1.8 mL of a freshly prepared FRAP reagent. The FRAP reagent was prepared by mixing 2.5 mL of a 10 mM solution of 2,4,6‐tris(2‐pyridyl)‐s‐triazine (TPTZ) in 40 mM hydrochloric acid with 2.5 mL of a 20 mM iron solution (chloride hexahydrate). This iron solution was dissolved in 25 mL of a 0.3 M acetate buffer at pH 3.6. Afterward, the extract solution was combined with the FRAP reagent and incubated at 37°C for 5 min. Subsequently, the absorbance of the resulting mixture was measured at a wavelength of 593 nm using a spectrophotometer. The reducing power of the ARE was determined by comparing the spectrophotometric absorbance of the sample to a standard curve generated from ferric sulfate. This comparative analysis facilitates the quantification of the extract's reducing capacity, thereby providing valuable insights into its antioxidant potential (Ou et al. 2002; Schwalm III et al. 2019).

#### 2.4.3 Total Flavonoid Content (TFC) and Total Phenolic Content (TPC) Measurement

The total flavonoid content (TFC) of the extracts was determined using the ammonium chloride calorimetric method. To perform the assay, 1 mL of the extract solution (containing 1 mg/mL quercetin) or quercetin (at concentrations ranging from 25 to 200 μg/mL) was mixed with 0.2 mL of a 10% (w/v) ammonium chloride solution in ethanol, 0.2 mL of 1 M potassium acetate, and 5.6 mL of distilled water. The resulting mixture was incubated for 30 min at room temperature. After the incubation period, the absorbance of the mixture was measured at 415 nm using a blank sample as a reference. The TFC was reported as milligrams of quercetin equivalent/g of dry extract (mgQE/g) (Behbahani et al. 2019).

The total phenolic content (TPC) of the extract was determined using the Folin–Ciocalteu reagent. In this procedure, 200 μL of the extract was mixed with 1 mL of Folin–Ciocalteu reagent, 5.8 mL of distilled water, and 3 mL of a 20% sodium carbonate solution. The mixture was stirred in a dark environment for 2 h, after which the absorbance was measured at 765 nm in triplicate. Gallic acid was used as the standard, and the TPC concentration was expressed as milligrams of gallic acid equivalent (GAE) per gram of extract (Falah et al. 2021).

#### 2.4.4 Gas Chromatography–Mass Spectrometry (GC/MS) Analysis

The GC/MS analysis of the ARE was performed using an Agilent 7890A mass spectrometer coupled to an Agilent 7000 gas chromatograph. For the analysis, a 1 mL aliquot of the extract was injected into the GC/MS apparatus (USA). The data obtained were analyzed using an HP5MS column, which measured 30 m in length, 0.25 mm in internal diameter, and had a film thickness of 0.25 μm. Helium gas was employed as the carrier gas for chromatographic separation. The temperature program for the oven during the extract analysis commenced at an initial temperature of 40°C, maintained for 2 min, followed by a gradual increase to 350°C at a rate of 5°C/min.

The constituents within the extract were identified by comparing their mass spectra with those cataloged in the National Institute of Standards and Technology (NIST) mass spectral library. This comparison facilitated the determination of the chemical compounds present in the extract (Keskes et al. 2017).

#### 2.4.5 Fourier Transform Infrared Spectroscopy (FT‐IR) Analysis

The chemical active compounds of the ARE were analyzed and evaluated using Fourier Transform Infrared (FT‐IR) spectroscopy in conjunction with potassium bromide (KBr) pellets. The FT‐IR analysis was conducted using a Shimadzu 8400S instrument, a product of Shimadzu Co., headquartered in Kyoto, Japan. During the analysis, the absorbance peaks of the sample were recorded within the spectral range of 4000 to 400 cm^−1^. This spectral range corresponds to the infrared wavelengths associated with the molecular vibrations of the compounds present in the extract. By analyzing the specific wavenumbers at which the absorbance peaks occur, researchers can gain insights into the functional groups and chemical bonds present within the sample (Shimadzu Co., Kyoto, Japan).

#### 2.4.6 Antibacterial Activity of ARE

Microbial strains were obtained from the Industrial Microbiology Laboratory at Ferdowsi University of Mashhad, Mashhad, Iran. The study employed the following strains: *L. innocua* (ATCC 33090), *Pseudomonas aeruginosa* (ATCC 2753), *E. coli* (ATCC 25922), *S. aureus* (ATCC 25923), and *Methicillin‐resistant* *Staphylococcus aureus* (MRSA) (ATCC 33591). It is imperative to emphasize that all necessary biosafety protocols were meticulously adhered to throughout the experimental procedures involving pathogenic strains. These measures included conducting experiments within a biosafety cabinet to minimize the risk of aerosolization and contamination. Appropriate personal protective equipment (PPE), such as gloves, lab coats, and masks, were utilized. Furthermore, the research team strictly adhered to established laboratory protocols for handling and disposing of biohazardous materials. Additionally, our research team complied with the guidelines set by the biosafety committee of our institution.

#### 2.4.7 Minimum Inhibitory Concentration (MIC) and Minimum Bactericidal Concentrations (MBC)

The MIC was determined by the microdilution method in 96 well microtitre plates. Briefly, fresh overnight bacterial cultures were standardized to 0.5 McFarland standards (1.5 × 10^8^ CFU/mL) using sterile phosphate‐buffered saline (PBS), with measurements taken via a spectrophotometer for each well. Various concentrations of ARE, ranging from 1.17 to 600 mg/mL were prepared in broth. A 20 μL suspension (1.5 × 10^8^ CFU/mL) of the microorganisms under investigation was introduced into each well, followed by the addition of 100 μL of the respective ARE concentrations. The positive control well contained both broth and microorganisms, while the negative control well consisted solely of broth. The microplate was incubated for 24 h at 37°C. Following this incubation period, approximately 10 μL of a 0.5% tetrazolium solution was added to each well, and the microplate was subsequently incubated at 37°C for an additional 20 min. The MIC was defined as the lowest concentration of ARE that inhibited visible growth of the indicator pathogens.

The minimum inhibitory concentration (MIC) of each extract was determined by transferring 10 μL from the MIC wells onto agar plates. These plates were subsequently incubated at 37°C for 24 h. The minimum bactericidal concentration (MBC) was identified as the lowest extract concentration that resulted in the complete eradication of viable bacteria. All experiments were conducted in triplicate (Alebooye et al. 2023; Ghasemi Pirbalouti et al. 2016).

#### 2.4.8 Well Diffusion Agar Assay

In Teles et al. (2021) investigation, pathogens were uniformly distributed on Mueller‐Hinton agar plates using a sterile spreader. Subsequently, four 6‐mm‐diameter wells were created on the agar surface using a sterile well cavity. Twenty microliters of ARE (at varying concentrations: 100, 200, 400, and 800 mg/mL) were introduced into each well. The plates were incubated at 37°C for 24 h to permit bacterial growth. Subsequently, the zones of inhibition surrounding each well were measured and documented in millimeters. These zones serve as indicators of the extent to which the ARE impeded the growth of the tested pathogens (Teles et al. 2021).

#### 2.4.9 Disk Diffusion Agar Test

The disk diffusion assay was conducted utilizing a 10 μL inoculum of a microbial suspension with a concentration of 1.5 × 10^8^ CFU/mL which was inoculated onto Mueller–Hinton agar. Discs containing varying concentrations of ARE (100, 200, 400, and 800 mg/mL) were placed on the surface of agar plates. The plates were subsequently incubated at 37°C for 24 h. Following incubation, the diameters of the inhibition zones were measured in millimeters. A chloramphenicol antibiotic disk (30 μg) was employed as a positive control in this experiment (Teles et al. 2021).

#### 2.4.10 Morphological Changes

To elucidate the morphological alterations induced in bacteria subjected to Antimicrobial Resistance (ARE) treatment, *Escherichia coli* and *S. aureus* cells were cultivated in the logarithmic growth phase. These cells were treated with ARE at a concentration of 2 μg of Inhibitory Concentration (MIC) and incubated at 37°C for 24 h. A control group, comprising untreated bacteria, was also included for comparative purposes. After the incubation period, the cells were harvested through centrifugation at 5000 × *g* for 5 min. To remove any remaining media or compounds, the cells underwent two washing cycles with a 0.1 M phosphate‐buffered saline (PBS) solution at a pH of 7.0. Subsequently, the cells were resuspended in PBS containing 2.5% glutaraldehyde and stored at 4°C overnight to facilitate fixation. This step ensures the preservation of cellular structures for further examination. In the subsequent phase, the cells underwent a series of water‐alcohol solutions with progressively increasing alcohol concentrations. Each dehydration step lasted for 10 min. The alcohol concentrations employed were 30%, 50%, 70%, 80%, 90%, and 100%. This gradual dehydration protocol is meticulously designed to effectively remove water from the cells while minimizing any potential damage. In the final phase, the dehydrated cells were thoroughly dried to eliminate any residual ethanol. The dried cells were subsequently coated with gold and analyzed using a scanning electron microscope (SEM). The specific SEM model utilized in this investigation was the Zeiss (LEO) 1450 VP model, manufactured in Germany (Lv et al. 2011; Rouhi et al. 2024).

#### 2.4.11 Cell Nucleic Acid Release

To evaluate the release of nucleic acids from bacterial cells, Lv et al. (2011) employed a methodology. Bacterial cells were harvested by centrifugation at 5000 × *g* for 10 min. Subsequently, the cells underwent three washing cycles to remove any residual media or compounds. They were then re‐suspended in a 0.1 M PBS solution at pH 7.0. To assess nucleic acid release, a 30 mL aliquot of the cell suspension was incubated with three distinct concentrations of ARE: a control group (without extract), the minimum inhibitory concentration (MIC), and twice the MIC. The incubation was conducted at 37°C with a shaking speed of 180 rpm for 1 h. After the incubation, 2 mL of the samples were collected and centrifuged at 12,000 × *g* for 2 min. This step separated the supernatant from the bacterial cells. The supernatant was subsequently analyzed for UV absorption at a wavelength of 260 nm, which serves as an indicator of nucleic acid release. The absorbance of the control cells, which were treated with PBS alone, was also measured at the same wavelength for comparative purposes. By comparing the absorbance values of the supernatant from the treated cells with those of the control cells, researchers can ascertain whether the ARE induced the release of nucleic acids from the bacterial cells (Lv et al. 2011).

#### 2.4.12 Cytotoxicity Assessment

The cytotoxicity of ARE was evaluated using the MTT assay. In this study, the Caco‐2 cell line, derived from human colon adenocarcinoma, was employed. The cells were seeded into 96‐well plates at a density of 3 to 5 × 10^3^ cells per well, utilizing 100 μL of Dulbecco's Modified Eagle Medium (DMEM) supplemented with 10% fetal bovine serum. Various concentrations of ARE, ranging from 0 to 600 mg/mL, were administered to each well in triplicate, specifically at concentrations of 0, 2.34, 4.68, 9.37, 18.75, 37.5, 75, 150, 300, and 600 mg/mL. Three wells were left untreated as a control group. Following a 48 h incubation period at 37°C, 10 μL of MTT solution (5 mg/mL in PBS) was introduced to each well. The MTT solution is metabolically reduced by viable cells, resulting in the production of a purple formazan compound. Subsequently, the cells were incubated for an additional 3 h. After this incubation, the supernatant was discarded, and 100 μL of dimethyl sulfoxide (DMSO) was added to each well to dissolve the formazan crystals generated by the viable cells. The plates were gently agitated to ensure complete dissolution. The absorbance of each well was then measured at 570 nm using a microtiter plate reader. This measurement quantifies the formazan product, which directly correlates with the number of viable cells. To determine cell viability, the absorbance at 570 nm of the treated cells is divided by the absorbance at 570 nm of the control cells, and the resulting value is multiplied by 100. This calculation yields the percentage of cell viability (Esghaei et al. 2018).

### 2.5 Statistical Analysis

Statistical analysis was conducted using SPSS version 20 software. One‐way analysis of variance (ANOVA) was employed to compare the measurement data among the different groups. A significance level of *p* < 0.05 was deemed statistically significant. To ensure accuracy and reliability, all measurements were performed in triplicate, thereby minimizing experimental variability and providing more robust results. Statistical analysis enables the examination of any significant differences between the groups, offering valuable insights into the effects of the variables under investigation.

## 3 Results and Discussion

### 3.1 Extraction Efficiency

The data presented in Table 1 demonstrates that the ultrasound extraction method surpasses the maceration method when employing a 6 g sample and 120 mL of solvent. These findings corroborate the findings of Safdar et al. (2017), who reported that 80% ethanol combined with ultrasound is the most effective method for extracting polyphenols from mango skin (Safdar et al. 2017). Similarly, Jacques et al. (2007) conducted a comparative study of ultrasound and maceration techniques for extracting compounds from *Ilex paraguariensis* leaves, concluding that ultrasound is a more straightforward, rapid, and effective approach for extracting organic compounds from plant materials compared to conventional maceration (Jacques et al. 2007). Furthermore, Ali et al. (2019) investigated the extraction efficiency of polyphenols from *Lycium barbarum* *L*. and discovered that ultrasound significantly enhanced extraction efficiency (Ali et al. 2019). Ultrasound's efficacy in plant extraction is attributed to its cavitation action. This action, achieved by applying an appropriate intensity, induces mechanical effects that irreversibly disrupt the plant membrane. This phenomenon, known as sonoporation, facilitates the effective delivery of materials (Yusoff et al. 2022).

**TABLE 1:** Extraction efficiency of *Aloe vera* rind extract by ultrasound and maceration.

Method	Initial weight (g)	Final weight (g)	Weight of the extract (g)	Extraction efficiency (%)
UAE	71.697 ± 0.005^A^	73.084 ± 0.007^A^	1.387 ± 0.008^A^	23.230 ± 0.007^A^
Maceration	68.735 ± 0.008^B^	69.761 ± 0.006^B^	1.026 ± 0.003^B^	17.160 ± 0.009^B^

*Note:* Data are expressed as the mean of triplicate ± SD. Uppercase letters in columns show significant difference at *p* < 0.05.

Abbreviation: UAE, Ultrasonic‐Assisted Extraction.

### 3.2 Comprehensive Studies of TPC and TFC and Antioxidant Activity

Plant materials exhibit substantial antioxidant properties, which are closely correlated with the presence of phenolic compounds. Generally, elevated concentrations of these compounds are associated with enhanced antioxidant activity (Hęś et al. 2019). Phenolic compounds and flavonoids, naturally occurring in plants, exert a pivotal role in mitigating lipid peroxidation by effectively scavenging reactive oxygen species and either reducing or chelating ferric ions within lipoxygenase. The disparity in antioxidant capacity observed among these compounds can be attributed to their remarkable ability to scavenge a diverse range of radicals, including DPPH free radicals and hydroxyl radicals (Behbahani et al. 2017).

The DPPH free radical is extensively employed to assess the primary antioxidant activity of a variety of substances, including pure antioxidant compounds, plant and fruit extracts, and food materials (Hęś et al. 2019). In the present study, the DPPH scavenging activity of the examined extract (ARE) was quantified at 2.375 ± 0.004 mg/mL and was compared to the reference compound BHT (Table 2). The reducing power of ARE, as evaluated through the DPPH assay, was found to be relatively high. However, as anticipated, BHT demonstrated a more significant reducing power in comparison to ARE, corroborating the findings of Solaberrieta et al. (2022). In agreement with our results, Mahadi et al. (2019) reported IC50 values of 113.18 μg/mL for ARE and 291.96 μg/mL for *Aloe vera* gel extract (Mahadi et al. 2019). Furthermore, Yahya et al. (2022) documented an IC50 value of 138.82 μg/mL for *Aloe vera* gel extract (Yahya et al. 2022). It is important to highlight that a lower IC50 value signifies a greater capacity of the sample to scavenge free radicals. The FRAP assay was utilized to evaluate the capacity of antioxidants to reduce ferric iron to its ferrous form. In this study, the FRAP value of the extract was determined to be 10.47 μmol Fe. Quispe et al. (2018) reported a value of 3.82 mM trolox/g fresh weight for *Aloe vera* . The FRAP and IC50 DPPH values for ARE were approximately 2.3 and 5.4 times greater than those of BHT, respectively, indicating the promising antioxidant potential of this plant (Vidic et al. 2014).

**TABLE 2:** TPC, TFC, DPPH scavenging activities and FRAP values of the *Aloe vera* rind extract.

Samples	TPC (mg GA/g)	TFC (mg QE/g)	DPPH (IC_50_ mg/mL)	FRAP (μmol Fe)
ARE	16.881 ± 0.001	32.083 ± 0.003	2.375 ± 0.004^A^	10.475 ± 0.005^A^
BHT	N/A	N/A	0.440 ± 0.002^B^	4.480 ± 0.003^B^

*Note:* Data are expressed as the mean of triplicate ± SD. TPC and TFC for BHT were not defined. Uppercase letters in columns show significant difference at *p* < 0.05. N/A: not applicable, as BHT is a synthetic antioxidant and does not contain natural phenolic or flavonoid compounds.

Abbreviations: GAE, gallic acid equivalents; QE, quercetin equivalents.

In the present investigation, the total phenolic content (TPC) of *Aloe vera* (ARE) was determined to be 16.881 ± 0.001 mg gallic acid equivalents (GAE) per gram of dry weight, while the total flavonoid content (TFC) was measured at 32.0857 mg quercetin equivalents (QE)/g of dry weight (Table 2). Vidic et al. (2014) analyzed the total phenolic and flavonoid profiles of ethanol extracts derived from the leaf peel and gel of various Aloe species. They reported that the peel extract exhibited the highest TPC at 7.99 mg GAE/g extract and the highest TFC at 9.17 mg QE/g extract (Vidic et al. 2014). Additionally, Solaberrieta et al. (2022) conducted 29 experiments on *Aloe vera* skin extract (AVSE) and found that the TPC values ranged from 86.5 to 125.8 mg GAE/g AVSE (Solaberrieta et al. 2022). Furthermore, Kaur et al. (2022) investigated the antioxidant properties of different parts of the *Aloe vera* plant and found that AVSE contained a significantly higher polyphenol content (1.12 ± 0.03 mg GAE/100 g dry weight) compared to *Aloe vera* gel extract (0.79 ± 0.03 mg GAE/100 g dry weight) (Kaur et al. 2022). The TPC of Aloe's leaf epidermis and flowers was reported to be 307.5 and 274.5 mg/100 g of lyophilized material, respectively (López et al. 2013). Collectively, these prior studies underscore the substantial antioxidant activity associated with the skin of *Aloe vera* leaves, corroborating the results obtained in our research (Añibarro‐Ortega et al. 2019; Solaberrieta et al. 2022).

The observed variability in the results can be attributed to several factors that influence the chemical composition and quality of plant extracts. These factors include the age and species of the plant, environmental conditions, drying techniques, and various extraction methods, all of which can significantly affect the chemical constituents present in the extracts (Hęś et al. 2019).

Scientific research has examined the impact of diverse extraction techniques on the antioxidant properties, total phenolic content (TPC), and total flavonoid content (TFC) in thyme plants. For instance, a comparative analysis of ultrasound‐assisted extraction and conventional extraction methods revealed that the extraction conditions substantially influence the levels of phenolic compounds, flavonoids, and carotenoids, as well as the overall antioxidant activity of the extracts (Munekata et al. 2020; Yusoff et al. 2022).

Phenolic compounds, including gallic acid, gentisic acid, catechin, quercetin, rutin, myricetin, and sinapic acid, constitute the primary phenolic constituents found in plants. These compounds are pivotal for antioxidant activity, as they inhibit the formation of free radicals, sequester iron, and prevent lipid oxidation (López et al. 2013).

### 3.3 GC/MS Analysis

**FIGURE 1:**
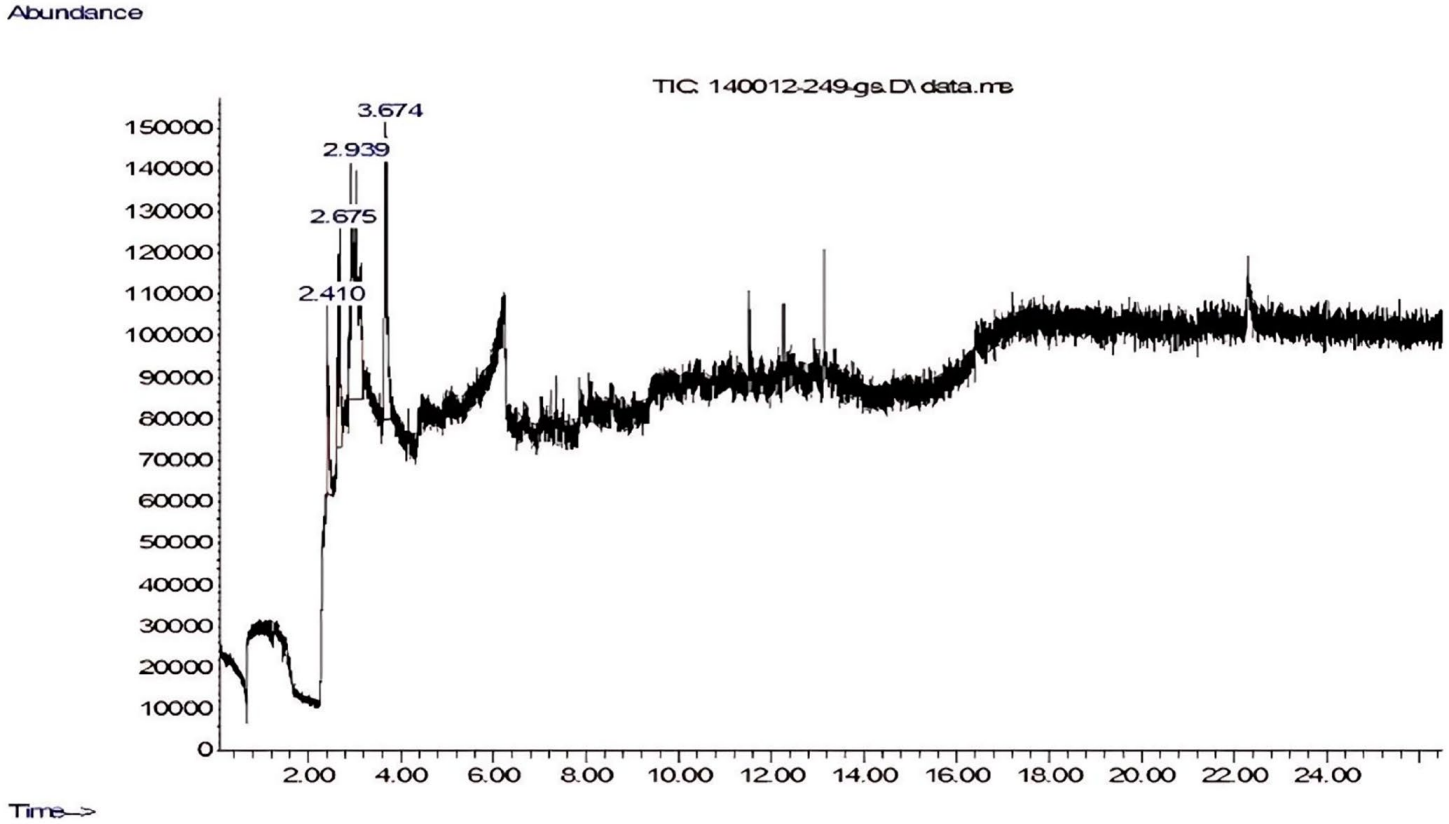
GC–MS chromatogram of *Aloe vera* rind extract.

The gas chromatography–mass spectrometry (GC–MS) analysis of the ARE revealed the presence of diverse bioactive compounds, including acids, alcohols, and ethers. As depicted in Figure 1, the chromatogram identified four distinct peaks corresponding to the following compounds: 2‐bromo‐4,5‐dimethoxycinnamic acid (11.9%), 1,4‐benzenediol (14.1%), 2,6‐bis(1,1‐dimethylethyl) (49.2%), and thieno[2,3‐c] furan‐3‐carbonitrile (24.7%), as detailed in Table 3. In a parallel study conducted by Brintha et al. (2017), GC–MS analysis of the methanolic extract from the leaves of *C. orchioides* also identified several compounds, including thieno[2,3‐c] furan‐3‐carbonitrile and 2‐amino‐4,6‐dihydro‐4,4,6,6‐tetramethyl, which exhibited a spectrum of pharmacological properties such as analgesic, antianginal, non‐opioid, antihypertensive, antiarthritic, and potential applications in dementia treatment and neurotransmitter uptake inhibition. Furthermore, derivatives of 3‐hydroxycinnamic acid have been detected in coffee extracts, which are prevalent in the cell walls of various plants, including those utilized for food purposes (Brintha et al. 2017). These compounds are synthesized through reactions within the shikimate pathway and constitute approximately one‐third of the phenolic derivatives that enhance the health‐promoting potential of food products (Medvedeva et al. 2022). Notably, the most abundant compounds identified in the ARE were di ester (2‐propylpentyl) and hexadecenoic acid ethyl ester of phthalic acid (Alghamdi et al. 2023).

**TABLE 3:** Identification of phytoconstituents isolated from *Aloe vera* rind extract.

No	Total name	Retention Time (min)	Type	Molecular formula	Molar mass (g/mol)	Percentage
1	2‐ Bromo‐4,5 dimethoxycinnamic acid	2.410	Acid	C_11_H_11_BrO_4_	287	11.967
2	1,4‐Benzenediol,2,6‐bis(1,1‐dimethylethyl)—	2.675	Alcohol	C_14_H_22_O_2_	222	14.100
3	Thieno[2,3‐c] furan‐3‐carbonitrile,2‐amino‐4,6‐dihydro‐4,4,6,6‐tetramethyl	2.939	Ether	C_11_H_14_N_2_OS	222	49.222
4	Pyrocatechol,3,5‐di‐tert‐butyl—	3.674	Alcohol	C_14_H_22_O_2_	222	24.710

In 2021, Shahinuzzaman et al. conducted research to optimize extraction conditions for *Ficus auriculata* fruit extract to maximize the yield of phenolic and bioactive compounds. Employing response surface methodology and ultrasonic‐assisted extraction techniques, the study established a strong correlation between experimental and anticipated values for antioxidant activity and total phenolic compounds (TPC). The optimized extraction parameters resulted in the highest observed DPPH radical scavenging activity and TPC. Liquid chromatography‐electrospray ionization‐mass spectrometry (LC–ESI–MS) profiling of the optimized extract revealed the presence of 18 bioactive compounds that may contribute to the antioxidant activity of *F. auriculata* fruits (Shahinuzzaman et al. 2021).

In a subsequent investigation conducted in 2020, Shahinuzzaman et al. assessed the TPC and antioxidant activity of latex from 18 cultivars of *Ficus carica* *L*. Various assays, including DPPH, ABTS, and FRAP, were employed to evaluate antioxidant activity. The bioactive compounds from *F. carica* latex were extracted using maceration and ultrasonic‐assisted extraction with 75% ethanol as the solvent. Among the cultivars studied, ‘White Genoa’ exhibited the highest antioxidant activity and TPC under identical extraction conditions. These findings suggest that *F. carica* latex possesses potential as a natural source of antioxidants, with implications for food supplements, additives, and pharmaceutical synthesis (Shahinuzzaman et al. 2020).

### 3.4 FTIR

**FIGURE 2:**
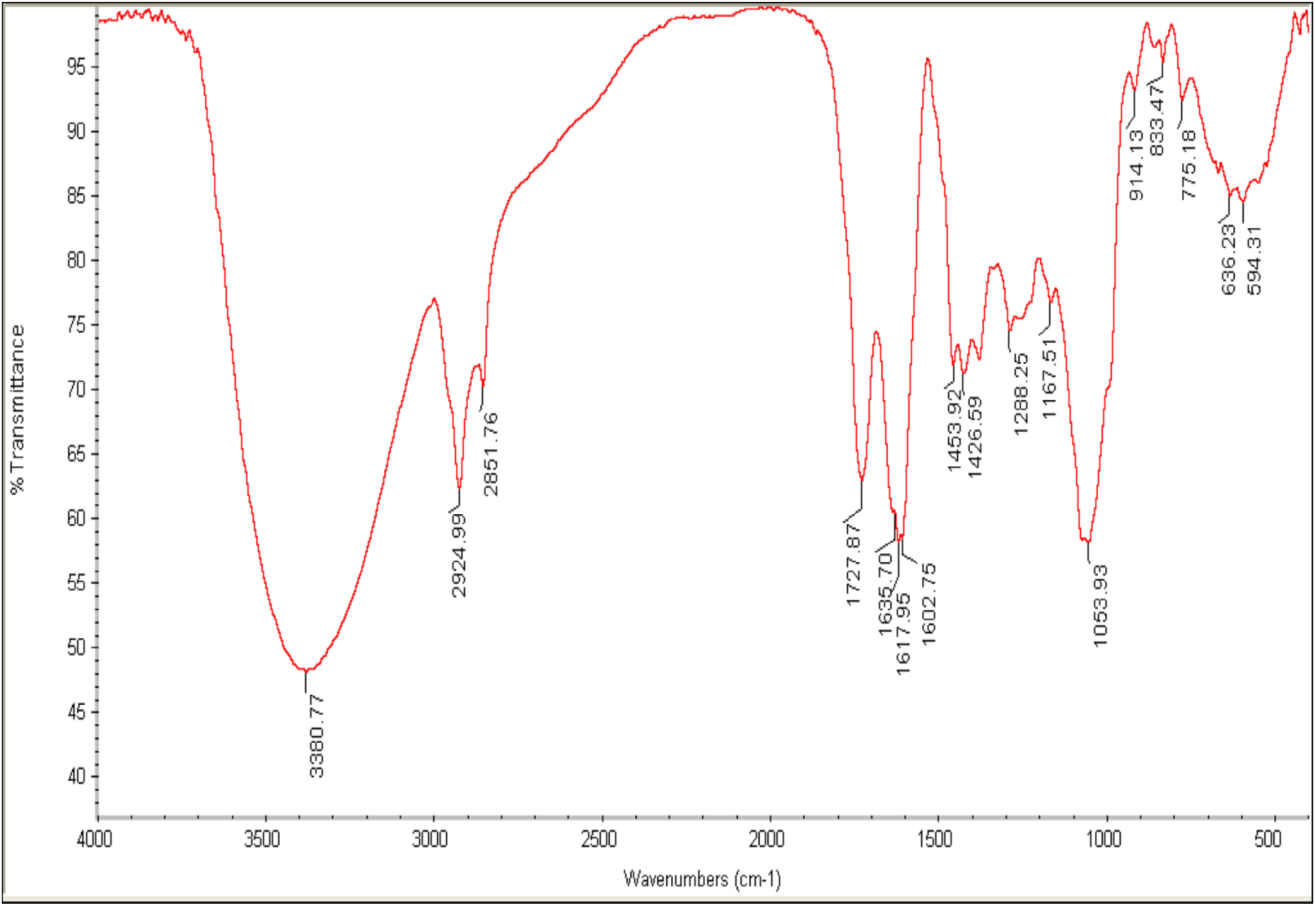
FTIR spectra of *Aloe vera* rind extract.

Figure 2 presents the Fourier transform infrared (FTIR) spectra of ARE. A prominent broad absorption peak at 3380.77 cm^−1^ is observed, which corresponds to the stretching of ‐OH groups. This indicates the presence of alcoholic compounds in ARE. The absorption band at 2924.99 cm^−1^ is characteristic of C‐H stretching, reflecting both symmetrical and asymmetrical C‐H stretching of aliphatic ‐CH and ‐CH2 groups. The presence of carbonyl compounds in ARE is confirmed by the absorption band at 1727.87 cm^−1^, which corresponds to C=O stretching. Peaks at approximately 1617.95 and 1426.59 cm^−1^ are attributed to the asymmetrical and symmetrical ‐COO stretching of carboxylate compounds, respectively. The absorption peak at 1288 cm^−1^ may indicate the presence of aliphatic compounds, specifically the C‐O‐C bond of aromatic acid esters and C‐OH groups of phenolic compounds. Additionally, the absorption peak at 1053.93 cm^−1^ is likely due to C‐O stretching, while the peak at 775 cm^−1^ is associated with C‐H stretching (Alizadeh Behbahani et al. 2020). ARE comprises a collection of hydroxyl, carboxyl, carbonyl, and functional groups associated with the phenolic ring. The spectral peaks observed in this investigation correspond to the presence of these constituents, aligning with findings from prior research (Fardsadegh and Jafarizadeh‐Malmiri 2019; Lim and Cheong 2015).

### 3.5 Antibacterial Activity

The study aimed to evaluate the antimicrobial properties of *Aloe vera* rind extract (ARE) employing both disk diffusion agar and well diffusion agar methodologies, as outlined in Tables 4 and 5. The data demonstrated a positive correlation between ARE concentration and the extent of inhibition halo, indicating its overall antimicrobial efficacy. Notably, *S. aureus* exhibited the greatest sensitivity to the extract across all concentrations tested, while *Pseudomonas aeruginosa* demonstrated the highest level of resistance.

**TABLE 4:** Results of well diffusion agar assays for *Aloe vera* rind extract against common food pathogens.

Bacteria	Well diffusion agar/concentration (mm)			
	100	200	400	800
*Listeria innocua*	7.10 ± 0.15^dD^	10.30 ± 0.27^cB^	13.70 ± 0.27^bB^	14.70 ± 0.25^aB^
Methicillin‐resistant *Staphylococcus aureus*	8.50 ± 0.40^dB^	9.60 ± 0.37^cC^	10.20 ± 0.27^bC^	11.20 ± 0.32^aC^
*Staphylococcus aureus*	9.10 ± 0.12^dA^	11.10 ± 0.18^cA^	16.50 ± 0.46^bA^	19.50 ± 0.50^aA^
*Escherichia coli*	7.80 ± 0.27^dC^	7.50 ± 0.52^cD^	10.10 ± 0.28^bC^	10.70 ± 0.25^aD^
*Pseudomonas aeruginosa*	0.00 ± 0.00^dE^	7.00 ± 0.16^cD^	8.10 ± 0.31^bD^	9.10 ± 0.26^aE^

*Note:* Lowercase letters in rows and uppercase letters in columns show a significant difference at *p* < 0.05.

**TABLE 5:** Results of disk diffusion agar assays for *Aloe vera* rind extract against common food pathogens.

Bacteria	Disk diffusion agar/concentration (mm)				
	100	200	400	800	Chloramphenicol
*Listeria innocua*	6.70 ± 0.47^cC^	9.00 ± 0.20^bB^	12.40 ± 0.50^aB^	13.20 ± 0.34^aB^	23.80 ± 0.21^dB^
Methicillin‐resistant *Staphylococcus aureus*	7.40 ± 0.16^cB^	7.60 ± 0.38^cD^	9.80 ± 0.28^bC^	11.70 ± 0.43^aC^	22.50 ± 0.36^dC^
*Staphylococcus aureus*	8.90 ± 0.31^dA^	9.80 ± 0.18^cA^	14.40 ± 0.60^bA^	18.70 ± 0.40^aA^	25.30 ± 0.17^eA^
*Escherichia coli*	7.36 ± 0.43^cB^	7.70 ± 0.25^bC^	9.10 ± 0.45^aD^	9.80 ± 0.31^aD^	21.50 ± 0.20^dD^
*Pseudomonas aeruginosa*	0.00 ± 0.00^bD^	0.00 ± 0.00^bE^	8.50 ± 0.45^aE^	9.10 ± 0.78^aD^	22.40 ± 0.23^cC^

*Note:* Lowercase letters in rows and uppercase letters in columns show a significant difference at *p* < 0.05.

Among the bacterial strains assessed, *S. aureus* exhibited the most significant average inhibition zone, measuring 19.50 ± 0.50 mm at a concentration of 800 mg/mL of ARE, as determined by the well diffusion agar method (Table 4). In contrast, *Pseudomonas aeruginosa* and *E. coli* exhibited the smallest inhibition zones. These findings suggest a direct correlation between ARE concentration and the magnitude of the inhibition zone, potentially attributed to the diverse biological compounds present in the *Aloe vera* rind, including catechin, sinapic acid, ercetin, quercitrin, rutin, myricetin, and epicatechin (López et al. 2013).

The ARE exhibited antibacterial properties against *S. aureus* , *Shigella* species, E.coli, and *Methicillin‐resistant Staphylococcus aureus* (MRSA), with the exception of *Salmonella typhimurium* , which demonstrated the highest level of resistance to both aqueous and ethanol extracts of ARE (Alghamdi et al. 2023).

The minimum inhibitory concentration (MIC) and minimum bactericidal concentration (MBC) results for the ARE against the tested microorganisms are presented in Table 6. The MIC values ranged from 18.75 to 75 mg/mL, while the MBC values ranged from 37.5 to 150 mg/mL. Notably, the growth of MRSA and *Pseudomonas aeruginosa* ( *P. aeruginosa* ) was inhibited at a concentration of 75 mg/mL, while *S. aureus* and *Lactobacillus innocua* were inhibited at 18.75 mg/mL, suggesting that these latter bacteria were the most susceptible. *Escherichia coli* was inhibited at 37.5 mg/mL. The MBC values for ARE against *Lactobacillus innocua*, *Pseudomonas aeruginosa* , *S. aureus* , *Escherichia coli* , and *Pseudomonas aeruginosa* were determined to be 37.5, 150, 37.5, 75, and 150 mg/mL, respectively.

**TABLE 6:** MIC and MBC of *Aloe vera* rind extract against test microorganisms.

Bacteria	MIC (mg/mL)	MBC (mg/mL)
*Listeria innocua*	18.75	37.5
Methicillin‐resistant *Staphylococcus aureus*	75	150
*Staphylococcus aureus*	18.75	37.5
*Escherichia coli*	37.5	75
*Pseudomonas aeruginosa*	75	150

To assess the antibacterial efficacy of *Aloe Vera* extract against pathogenic bacteria, specifically *E. coli* , *P. aeruginosa* , and *Bacillus cereus* ( *B. cereus* ), varying concentrations of 40, 80, and 120 mg/mL were employed through the agar well diffusion method. Both aqueous and ethanol extracts were utilized in this investigation. A study conducted by Nsofor et al. (2023) demonstrated that ethanol extracts exhibited substantial inhibitory activity. The maximum inhibition was observed for *E. coli* (averaging 11.3 ± 1.52 to 21.6 ± 2.08 mm), *P. aeruginosa* (15.6 ± 1.15 to 23.6 ± 0.58 mm), and *B. cereus* (15.6 ± 1.52 to 19.3 ± 1.52 mm). The minimum inhibitory concentration (MIC) for the aqueous extracts against the tested organisms ranged from 12.5 to 25 mg/mL, while the MIC for the ethanol extract was between 6.25 and 12.5 mg/mL. Similarly, the minimum bactericidal concentration (MBC) for the aqueous extract varied from 25 to 50 mg/mL, and for the ethanol extract, it ranged from 12.5 to 25 mg/mL. These findings suggest that *Aloe Vera* gel extract, particularly in its ethanol form, may be an effective antibacterial agent against human pathogens for medicinal, cosmetic, and food applications. Furthermore, a study conducted by Yebpella et al. (2011) revealed that the aqueous extract from the green rind and gel of *Aloe vera* exhibited significant activity against the tested bacteria. The minimum inhibitory concentration (MIC) values ranged from 6.25 to 25 mg/mL, while the minimum bactericidal concentration (MBC) values ranged from 12.5 to 50 mg/mL. These findings align with our own observations (Falah et al. 2021; Nsofor et al. 2023; Yebpella et al. 2011).

The antimicrobial properties of the aqueous extract of *Aloe vera* (ARE) are attributed to its active phytochemical constituents. Phytochemical analysis of the ARE revealed the presence of tannins and flavonoids, both of which are known for their antimicrobial effects. Tannins, for instance, inhibit protein synthesis in cells by forming irreversible complexes with proline proteins (Behbahani et al. 2017). Furthermore, anthraquinone, another active compound found in *Aloe vera* leaves, has been associated with antimicrobial activity and immune system modulation. It functions by obstructing the ribosomal A site, which serves as the entry point for aminoacylated tRNA, thereby inhibiting bacterial protein synthesis (Pandey and Mishra 2010; Pugh et al. 2001).

### 3.6 Morphological Changes

**FIGURE 3:**
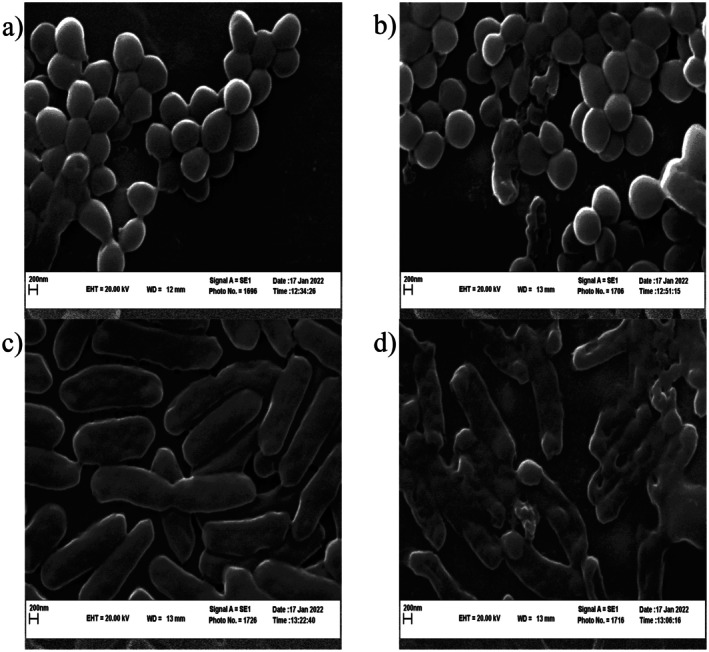
SEM of *S. aureus* cells: (a) untreated (magnification ×2000) and (b) treated with the *Aloe vera* rind extract and *Escherichia coli* cells: (c) untreated (magnification ×2000) and (d) treated with the *Aloe vera* rind extract at 2MIC value for 24 h (magnification ×2000).

*S. aureus* and *E. coli* , the most representative gram‐positive and gram‐negative bacteria, respectively, were selected for examination of the plant extract's impact on their surface morphology using scanning electron microscopy (SEM). Microstructural analyses indicated that the extract enhanced cell permeability and compromised membrane integrity. As depicted in Figure 3b,d bacteria cells treated with the extract at a concentration of 2 micrograms per milliliter (MIC) exhibited significant deformation, resulting in altered and incomplete cellular shapes. In contrast, Figure 3a,c illustrate the normal morphology of *S. aureus* and *E. coli* cells, respectively. These findings support the antibacterial properties of the extract, corroborating previous research in this field (Añibarro‐Ortega et al. 2019).

### 3.7 Release of Cell Nucleic Acids

The data presented in Table 7 indicates that increasing the extract concentration compared to the control group results in a heightened release of cellular nucleic acids, suggesting a greater extent of bacterial cell destruction. Among the evaluated bacterial strains, *S. aureus* exhibited the highest absorbance at 2 MIC at a wavelength of 260 nm (1.992 ± 0.002) (*p* < 0.05), indicating an irreversible disruption of the cytoplasmic membrane of the bacterial cells. These findings align with the outcomes of the antibacterial assays conducted. A potential morphological alteration observed is the exodus of nucleic acids from the cell wall into the external environment. Elevated concentrations of phenolic compounds may facilitate an increased release of intracellular components by modifying the cell membrane, thereby enhancing its permeability. These alterations could also impact essential membrane functions crucial for cellular exchange and the synthesis of metabolites (Ben Othman et al. 2017).

**TABLE 7:** The effect of *Aloe vera* rind extract on release of Cell nucleic acids (OD260 nm) of the tested microorganisms.

	ARE concentration (mg/mL)		
Microorganism	0	MIC	2MIC
*Staphylococcus aureus*	0.003 ± 0.050^cA^	0.639 ± 0.070^bA^	1.992 ± 0.090^aA^
*Listeria innocua*	0.002 ± 0.040^cB^	0.621 ± 0.080^bB^	1.476 ± 0.130^aB^
*Escherichia coli*	0.003 ± 0.080^cA^	0.279 ± 0.160^bD^	1.438 ± 0.120^aC^
Methicillin‐resistant *Staphylococcus aureus*	0.002 ± 0.020^cB^	0.463 ± 0.140^bC^	1.279 ± 0.150^aD^
*Pseudomonas aeruginosa*	0.001 ± 0.100^cC^	0.165 ± 0.130^bE^	0.541 ± 0.070^aE^

*Note:* Values represent means of three independent replicates ± SD. Small letters have been used for comparison of ARE concentrations in each bacterium (row) and capital letters have been used for comparison of bacteria in each ARE concentration (column) (*p* < 0.05) (one‐way ANOVA with Tukey‘s HSD post‐hoc test).

### 3.8 Cytotoxicity Assessment

Previous studies have demonstrated the cytotoxic activity of plants and their derivatives, particularly in cancer cell lines, underscoring their potential therapeutic applications (Wang et al. 2007). These plants are rich in bioactive components that offer health benefits and are commonly incorporated into gourmet cuisine. Notably, certain plant‐derived bioactive components have been unequivocally confirmed to possess anti‐cancer properties.

**FIGURE 4:**
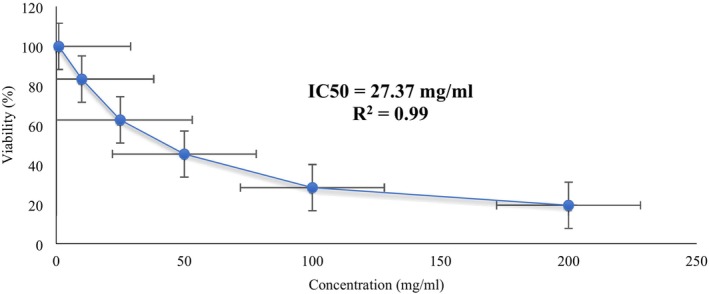
Cytotoxic activity of *Aloe vera* rind extract against Caco‐2 cell lines.

In our study, we investigated the impact of the plant extract (ARE) on the proliferation of Caco‐2 cell lines using the MTT assay. We evaluated concentrations ranging from 0 to 600 mg/mL and constructed a dose–response curve. The curve exhibited a progressive decline in the number of viable cells with escalating concentrations of ARE. At a concentration of 600 mg/mL, only 6.6% of the cells remained viable (Figure 4). Employing Excel Software, we determined the IC50 value (27.37 ± 0.10 mg/mL) and *R* ^2^ value (0.99), indicating a consistent augmentation in cytotoxicity with higher concentrations of ARE.

Parallel findings have been reported by Majumder et al. (2020), who established an IC50 value of 23 μg/mL for *Aloe vera* leaf extract against the breast cancer cell line (MCF‐7) (Majumder et al. 2020). Shalabi et al. (2015) conducted an in vitro study to assess the anticancer effects of *Aloe vera* and Calligonum comosum extracts against hepatocellular carcinoma (HepG2) cells and reported IC50 values of 10.45 ± 0.31 and 9.60 ± 0.01 μg/mL, respectively (Shalabi et al. 2015).

Antioxidant compounds play a pivotal role in cytotoxicity by reacting with free radicals and chelating toxic compounds and metals. A direct correlation exists between antioxidant and cytotoxic effects: an increase in the concentration of antioxidant compounds, such as polyphenols, carotenoids, and tannins, leads to a heightened cytotoxic effect (Hęś et al. 2019; López et al. 2013).

## 4 Conclusion

In our study, we compared ultrasound extraction to maceration as a more efficient method for extracting active compounds from a substance called ARE. The results demonstrated that ultrasound extraction yielded a richer extract, containing a primary chemical constituent known as Thieno [2,3‐c] furan‐3‐carbonitrile,2‐amino‐4,6‐dihydro‐4,4,6,6‐tetramethyl. This compound accounted for 49.2% of the ARE extract. ARE exhibited robust antioxidant and antimicrobial properties, particularly against gram‐positive bacteria. Furthermore, we observed a dose‐dependent cytotoxic effect on Caco‐2 cell lines when exposed to ARE at varying concentrations ranging from 0 to 600 mg/mL for a duration of 48 h. These findings suggest that ARE possesses potential therapeutic applications in the food, pharmaceutical, and cosmetic industries. Additionally, its utilization could contribute to the principles of a circular economy by reducing food waste and mitigating environmental consequences. Nevertheless, further research is necessary to elucidate the underlying mechanisms of ARE's anticancer activity and facilitate its widespread adoption across diverse industries.

## Author Contributions

**Fahime Lavi Arab:** data curation (equal), investigation (equal), writing – original draft (equal), writing – review and editing (equal). **Alireza Vasiee:** data curation (equal), investigation (equal), writing – original draft (equal), writing – review and editing (equal). **Farideh Tabatabaee Yazdi:** conceptualization (equal), formal analysis (equal), methodology (equal), project administration (equal), supervision (equal), validation (equal), writing – original draft (equal), writing – review and editing (equal).

## Conflicts of Interest

The authors declare no conflicts of interest.

## Data Availability

All data generated or analyzed during this study is included in this published article.

## References

[fsn371264-bib-0001] Ahmad Nejhad, A. , B. Alizadeh Behbahani , M. Hojjati , A. Vasiee , and M. A. Mehrnia . 2023. “Identification of Phytochemical, Antioxidant, Anticancer and Antimicrobial Potential of *Calotropis procera* Leaf Aqueous Extract.” Scientific Reports 13, no. 1: 14716. 37679486 10.1038/s41598-023-42086-1 PMC10485245

[fsn371264-bib-0002] Alebooye, P. , F. Falah , A. Vasiee , F. T. Yazdi , and S. A. Mortazavi . 2023. “Spent Coffee Grounds as a Potential Culture Medium for γ‐Aminobutyric Acid (GABA) Production by Levilactobacillus Brevis PML1.” LWT 189: 115553.

[fsn371264-bib-0003] Alghamdi, A. , W. Alshehri , B. Sajer , et al. 2023. “Biological Activities and GC‐MS Analysis of Aloe Vera and *Opuntia ficus‐indica* Extracts.” Journal of Chemistry 2023, no. 1: 6504505.

[fsn371264-bib-0004] Ali, M. C. , J. Chen , H. Zhang , Z. Li , L. Zhao , and H. Qiu . 2019. “Effective Extraction of Flavonoids From *Lycium barbarum* L. Fruits by Deep Eutectic Solvents‐Based Ultrasound‐Assisted Extraction.” Talanta 203: 16–22. 31202321 10.1016/j.talanta.2019.05.012

[fsn371264-bib-0005] Alizadeh Behbahani, B. , F. Falah , F. Lavi Arab , M. Vasiee , and F. Tabatabaee Yazdi . 2020. “Chemical Composition and Antioxidant, Antimicrobial, and Antiproliferative Activities of *Cinnamomum zeylanicum* Bark Essential Oil.” Evidence‐Based Complementary and Alternative Medicine 2020, no. 1: 5190603. 32419807 10.1155/2020/5190603 PMC7210559

[fsn371264-bib-0006] Alizadeh Behbahani, B. , F. Tabatabaei Yazdi , S. A. Mortazavi , F. Zendeboodi , M. M. Gholian , and A. Vasiee . 2013. “Effect of Aqueous and Ethanolic Extract of *Eucalyptus camaldulensis* L. on Food Infection and Intoxication Microorganisms In Vitro.” Journal of Paramedical Sciences 4: 3.

[fsn371264-bib-0007] Añibarro‐Ortega, M. , J. Pinela , L. Barros , et al. 2019. “Compositional Features and Bioactive Properties of *Aloe vera* Leaf (Fillet, Mucilage, and Rind) and Flower.” Antioxidants 8, no. 10: 444. 31581507 10.3390/antiox8100444 PMC6826699

[fsn371264-bib-0008] Arbab, S. , H. Ullah , W. Weiwei , et al. 2021. “Comparative Study of Antimicrobial Action of Aloe Vera and Antibiotics Against Different Bacterial Isolates From Skin Infection.” Veterinary Medicine and Science 7, no. 5: 2061–2067. 33949142 10.1002/vms3.488 PMC8464272

[fsn371264-bib-0009] Behbahani, B. A. , M. Noshad , and F. Falah . 2019. “Study of Chemical Structure, Antimicrobial, Cytotoxic and Mechanism of Action of *Syzygium aromaticum* Essential Oil on Foodborne Pathogens.” Potravinarstvo Slovak Journal of Food Sciences 13, no. 1: 875–883.

[fsn371264-bib-0010] Behbahani, B. A. , F. Shahidi , F. T. Yazdi , S. A. Mortazavi , and M. Mohebbi . 2017. “Use of *Plantago major* Seed Mucilage as a Novel Edible Coating Incorporated With *Anethum graveolens* Essential Oil on Shelf Life Extension of Beef in Refrigerated Storage.” International Journal of Biological Macromolecules 94: 515–526. 27771410 10.1016/j.ijbiomac.2016.10.055

[fsn371264-bib-0011] Behbahani, B. A. , F. T. Yazdi , A. Mortazavi , M. M. Gholian , F. Zendeboodi , and A. Vasiee . 2014. “Antimicrobial Effect of Carboxy Methyl Cellulose (CMC) Containing Aqueous and Ethanolic *Eucalyptus camaldulensis* L. Leaves Extract Against *Streptococcus pyogenes* , Pseudomonas Aeruginosa and *Staphylococcus epidermidis* .” Archives of Advances in Biosciences 5: 2.

[fsn371264-bib-0012] Behbahani, B. A. , F. T. Yazdi , F. Shahidi , H. Noorbakhsh , A. Vasiee , and A. Alghooneh . 2018. “Phytochemical Analysis and Antibacterial Activities Extracts of Mangrove Leaf Against the Growth of Some Pathogenic Bacteria.” Microbial Pathogenesis 114: 225–232. 29208540 10.1016/j.micpath.2017.12.004

[fsn371264-bib-0013] Behbahani, B. A. , F. T. Yazdi , A. Vasiee , and S. A. Mortazavi . 2018. “Oliveria Decumbens Essential Oil: Chemical Compositions and Antimicrobial Activity Against the Growth of Some Clinical and Standard Strains Causing Infection.” Microbial Pathogenesis 114: 449–452. 29241765 10.1016/j.micpath.2017.12.033

[fsn371264-bib-0014] Ben Othman, M. , K. Bel Hadj Salah‐Fatnassi , S. Ncibi , A. Elaissi , and L. Zourgui . 2017. “Antimicrobial Activity of Essential Oil and Aqueous and Ethanol Extracts of *Teucrium polium* L. Subsp. Gabesianum (LH) From Tunisia.” Physiology and Molecular Biology of Plants 23: 723–729. 28878510 10.1007/s12298-017-0444-9 PMC5567705

[fsn371264-bib-0015] Brintha, S. , S. Rajesh , R. Renuka , V. Santhanakrishnan , and R. Gnanam . 2017. “Phytochemical Analysis and Bioactivity Prediction of Compounds in Methanolic Extracts of *Curculigo orchioides* Gaertn.” Journal of Pharmacognosy and Phytochemistry 6, no. 4: 192–197.

[fsn371264-bib-0016] Chaudhary, A. , N. Kumar , R. Kumar , and R. K. Salar . 2019. “Antimicrobial Activity of Zinc Oxide Nanoparticles Synthesized From *Aloe vera* Peel Extract.” SN Applied Sciences 1: 1–9.

[fsn371264-bib-0017] Esghaei, M. , H. Ghaffari , B. R. Esboei , Z. E. Tapeh , F. B. Salim , and M. Motevalian . 2018. “Evaluation of Anticancer Activity of *Camellia sinensis* in the Caco‐2 Colorectal Cancer Cell Line.” Asian Pacific Journal of Cancer Prevention: APJCP 19, no. 6: 1697. 29938468 10.22034/APJCP.2018.19.6.1697 PMC6103574

[fsn371264-bib-0018] Falah, F. , K. Shirani , A. Vasiee , F. T. Yazdi , and B. A. Behbahani . 2021. “In Vitro Screening of Phytochemicals, Antioxidant, Antimicrobial, and Cytotoxic Activity of Echinops Setifer Extract.” Biocatalysis and Agricultural Biotechnology 35: 102102.

[fsn371264-bib-0019] Fardsadegh, B. , and H. Jafarizadeh‐Malmiri . 2019. “ *Aloe vera* Leaf Extract Mediated Green Synthesis of Selenium Nanoparticles and Assessment of Their In Vitro Antimicrobial Activity Against Spoilage Fungi and Pathogenic Bacteria Strains.” Green Processing and Synthesis 8, no. 1: 399–407.

[fsn371264-bib-0020] Faria, G. , M. Souza , J. Oliveira , C. Costa , M. Collares , and C. Prentice . 2020. “Effect of Ultrasound‐Assisted Cold Plasma Pretreatment to Obtain Sea Asparagus Extract and Its Application in Italian Salami.” Food Research International 137: 109435. 33233116 10.1016/j.foodres.2020.109435

[fsn371264-bib-0021] Ghasemi Pirbalouti, A. , A. Izadi , F. Malek Poor , and B. Hamedi . 2016. “Chemical Composition, Antioxidant and Antibacterial Activities of Essential Oils From *Ferulago angulata* .” Pharmaceutical Biology 54, no. 11: 2515–2520. 27102982 10.3109/13880209.2016.1162816

[fsn371264-bib-0022] Ghazanfari, N. , S. Fallah , A. Vasiee , and F. T. Yazdi . 2023. “Optimization of Fermentation Culture Medium Containing Food Waste for l‐Glutamate Production Using Native Lactic Acid Bacteria and Comparison With Industrial Strain.” LWT‐ Food Science and Technology 184: 114871.

[fsn371264-bib-0023] Guo, X. , and N. Mei . 2016. “ *Aloe vera* : A Review of Toxicity and Adverse Clinical Effects.” Journal of Environmental Science and Health, Part C 34, no. 2: 77–96. 10.1080/10590501.2016.1166826 PMC6349368 26986231

[fsn371264-bib-0024] Hęś, M. , K. Dziedzic , D. Górecka , A. Jędrusek‐Golińska , and E. Gujska . 2019. “ *Aloe vera* (L.) Webb.: Natural Sources of Antioxidants–A Review.” Plant Foods for Human Nutrition 74: 255–265. 31209704 10.1007/s11130-019-00747-5 PMC6684795

[fsn371264-bib-0025] Jacques, R. A. , L. dos Santos Freitas , V. F. Pérez , et al. 2007. “The Use of Ultrasound in the Extraction of *Ilex paraguariensis* Leaves: A Comparison With Maceration.” Ultrasonics Sonochemistry 14, no. 1: 6–12. 16439181 10.1016/j.ultsonch.2005.11.007

[fsn371264-bib-0026] Jalil Sarghaleh, S. , B. Alizadeh Behbahani , M. Hojjati , A. Vasiee , and M. Noshad . 2023. “Evaluation of the Constituent Compounds, Antioxidant, Anticancer, and Antimicrobial Potential of Prangos Ferulacea Plant Extract and Its Effect on *Listeria monocytogenes* Virulence Gene Expression.” Frontiers in Microbiology 14: 1202228. 37492261 10.3389/fmicb.2023.1202228 PMC10364450

[fsn371264-bib-0027] Kaur, R. , S. Willis , L. Shackelford , L. Walker , and M. Verghese . 2022. “Determination of Antioxidant Potential of Selected Parts of *Aloe vera* Plant.” Journal of Food & Nutritional Sciences 4, no. 2: 2050–2067.

[fsn371264-bib-0028] Keskes, H. , S. Belhadj , L. Jlail , et al. 2017. “LC‐MS–MS and GC‐MS Analyses of Biologically Active Extracts and Fractions From Tunisian *Juniperus phoenice* Leaves.” Pharmaceutical Biology 55, no. 1: 88–95. 27925471 10.1080/13880209.2016.1230139 PMC7011873

[fsn371264-bib-0029] Lim, Z. X. , and K. Y. Cheong . 2015. “Effects of Drying Temperature and Ethanol Concentration on Bipolar Switching Characteristics of Natural *Aloe vera* ‐Based Memory Devices.” Physical Chemistry Chemical Physics 17, no. 40: 26833–26853. 26400096 10.1039/c5cp04622j

[fsn371264-bib-0030] López, A. , M. S. De Tangil , O. Vega‐Orellana , A. S. Ramírez , and M. Rico . 2013. “Phenolic Constituents, Antioxidant and Preliminary Antimycoplasmic Activities of Leaf Skin and Flowers of *Aloe vera* (L.) Burm. f.(Syn. *A. barbadensis* Mill.) From the Canary Islands (Spain).” Molecules 18, no. 5: 4942–4954. 23624648 10.3390/molecules18054942 PMC6270129

[fsn371264-bib-0031] Lv, F. , H. Liang , Q. Yuan , and C. Li . 2011. “In Vitro Antimicrobial Effects and Mechanism of Action of Selected Plant Essential Oil Combinations Against Four Food‐Related Microorganisms.” Food Research International 44, no. 9: 3057–3064.

[fsn371264-bib-0032] Mahadi, S. B. , R. A. S. Handayani , W. Widowati , et al. 2019. “Antioxidant and Anti‐Tyrosinase Activities of *Aloe vera* Rind and Gel Extracts.” Global Medical & Health Communication 7, no. 3: 170–176.

[fsn371264-bib-0033] Majumder, R. , P. Parida , S. Paul , and P. Basak . 2020. “In Vitro and In Silico Study of *Aloe vera* Leaf Extract Against Human Breast Cancer.” Natural Product Research 34, no. 16: 2363–2366. 30600703 10.1080/14786419.2018.1534848

[fsn371264-bib-0034] Medvedeva, M. , N. Kitsilovskaya , Y. Stroylova , I. Sevostyanova , A. A. Saboury , and V. Muronetz . 2022. “Hydroxycinnamic Acid Derivatives From Coffee Extracts Prevent Amyloid Transformation of Alpha‐Synuclein.” Biomedicine 10, no. 9: 2255. 10.3390/biomedicines10092255 PMC9496549 36140356

[fsn371264-bib-0035] Munekata, P. E. S. , G. Rocchetti , M. Pateiro , L. Lucini , R. Domínguez , and J. M. Lorenzo . 2020. “Addition of Plant Extracts to Meat and Meat Products to Extend Shelf‐Life and Health‐Promoting Attributes: An Overview.” Current Opinion in Food Science 31: 81–87.

[fsn371264-bib-0036] Negahdari, S. , H. Galehdari , M. Kesmati , A. Rezaie , and G. Shariati . 2017. “Wound Healing Activity of Extracts and Formulations of *Aloe vera* , Henna, Adiantum Capillus‐Veneris, and Myrrh on Mouse Dermal Fibroblast Cells.” International Journal of Preventive Medicine 8, no. 1: 18. 28382194 10.4103/ijpvm.IJPVM_338_16 PMC5364744

[fsn371264-bib-0037] Nsofor, O. U. , A. P. Ogochukwu , A. O. Gabriel , E. Emmanuel , and C. M. Linus . 2023. “Evaluation of Antibacterial Activity of *Aloe vera* Extract on Some Bacterial Pathogens.” International Journal of Phytology Research 3, no. 1: 26–29.

[fsn371264-bib-0038] Okoh, S. O. , O. T. Asekun , O. B. Familoni , and A. J. Afolayan . 2014. “Antioxidant and Free Radical Scavenging Capacity of Seed and Shell Essential Oils Extracted From *Abrus precatorius* (L).” Antioxidants 3, no. 2: 278–287. 26784871 10.3390/antiox3020278 PMC4665479

[fsn371264-bib-0039] Ou, B. , D. Huang , M. Hampsch‐Woodill , J. A. Flanagan , and E. K. Deemer . 2002. “Analysis of Antioxidant Activities of Common Vegetables Employing Oxygen Radical Absorbance Capacity (ORAC) and Ferric Reducing Antioxidant Power (FRAP) Assays: A Comparative Study.” Journal of Agricultural and Food Chemistry 50, no. 11: 3122–3128. 12009973 10.1021/jf0116606

[fsn371264-bib-0040] Pandey, R. , and A. Mishra . 2010. “Antibacterial Activities of Crude Extract of *Aloe barbadensis* to Clinically Isolated Bacterial Pathogens.” Applied Biochemistry and Biotechnology 160: 1356–1361. 19263248 10.1007/s12010-009-8577-0

[fsn371264-bib-0041] Pugh, N. , S. A. Ross , M. A. ElSohly , and D. S. Pasco . 2001. “Characterization of Aloeride, a New High‐Molecular‐Weight Polysaccharide From *Aloe vera* With Potent Immunostimulatory Activity.” Journal of Agricultural and Food Chemistry 49, no. 2: 1030–1034. 11262067 10.1021/jf001036d

[fsn371264-bib-0042] Quispe, C. , M. Villalobos , J. Bórquez , and M. Simirgiotis . 2018. “Chemical Composition and Antioxidant Activity of *Aloe vera* From the Pica Oasis (Tarapacá, Chile) by UHPLC‐Q/Orbitrap/MS/MS.” Journal of Chemistry 2018, no. 1: 6123850.

[fsn371264-bib-0043] Radha, M. H. , and N. P. Laxmipriya . 2015. “Evaluation of Biological Properties and Clinical Effectiveness of Aloeávera: áAásystematic Review.” Journal of Traditional and Complementary Medicine 5, no. 1: 21–26. 26151005 10.1016/j.jtcme.2014.10.006 PMC4488101

[fsn371264-bib-0044] Rostami, H. , and S. M. T. Gharibzahedi . 2016. “Microwave‐Assisted Extraction of Jujube Polysaccharide: Optimization, Purification and Functional Characterization.” Carbohydrate Polymers 143: 100–107. 27083348 10.1016/j.carbpol.2016.01.075

[fsn371264-bib-0045] Rouhi, A. , F. Falah , M. Azghandi , et al. 2024. “Investigating the Effect of *Lactiplantibacillus plantarum* TW57‐4 in Preventing Biofilm Formation and Expression of Virulence Genes in *Listeria monocytogenes* ATCC 19115.” LWT‐ Food Science and Technology 191: 115669.

[fsn371264-bib-0046] Safdar, M. N. , T. Kausar , and M. Nadeem . 2017. “Comparison of Ultrasound and Maceration Techniques for the Extraction of Polyphenols From the Mango Peel.” Journal of Food Processing and Preservation 41, no. 4: e13028.

[fsn371264-bib-0047] Schwalm, N. D., III , W. Mojadedi , E. S. Gerlach , M. Benyamin , M. A. Perisin , and K. L. Akingbade . 2019. “Developing a Microbial Consortium for Enhanced Metabolite Production From Simulated Food Waste.” Fermentation 5, no. 4: 98.

[fsn371264-bib-0048] Shahinuzzaman, M. , P. Akhtar , N. Amin , et al. 2021. “New Insights of Phenolic Compounds From Optimized Fruit Extract of *Ficus auriculata* .” Scientific Reports 11, no. 1: 12503. 34127747 10.1038/s41598-021-91913-w PMC8203732

[fsn371264-bib-0049] Shahinuzzaman, M. , Z. Yaakob , F. H. Anuar , et al. 2020. “In Vitro Antioxidant Activity of *Ficus carica* L. Latex From 18 Different Cultivars.” Scientific Reports 10, no. 1: 10852. 32616768 10.1038/s41598-020-67765-1 PMC7331616

[fsn371264-bib-0050] Shalabi, M. , K. Khilo , M. M. Zakaria , M. G. Elsebaei , W. Abdo , and W. Awadin . 2015. “Anticancer Activity of *Aloe vera* and *Calligonum comosum* Extracts Separetely on Hepatocellular Carcinoma Cells.” Asian Pacific Journal of Tropical Biomedicine 5, no. 5: 375–381.

[fsn371264-bib-0051] Solaberrieta, I. , A. Jiménez , and M. C. Garrigós . 2022. “Valorization of *Aloe vera* Skin by‐Products to Obtain Bioactive Compounds by Microwave‐Assisted Extraction: Antioxidant Activity and Chemical Composition.” Antioxidants 11, no. 6: 1058. 35739955 10.3390/antiox11061058 PMC9220353

[fsn371264-bib-0052] Subramaniam, P. , S. Dwivedi , E. Uma , and K. G. Babu . 2012. “Effect of Pomegranate and *Aloe Vera* Extract on *Streptococcus mutans*: An: In Vitro: Study.” Dental Hypotheses 3, no. 3: 99–105.

[fsn371264-bib-0053] Teles, A. M. , J. V. Silva‐Silva , J. M. P. Fernandes , et al. 2021. “GC‐MS Characterization of Antibacterial, Antioxidant, and Antitrypanosomal Activity of *Syzygium aromaticum* Essential Oil and Eugenol.” Evidence‐Based Complementary and Alternative Medicine 2021, no. 1: 6663255. 33688364 10.1155/2021/6663255 PMC7914077

[fsn371264-bib-0054] Vidic, D. , E. Tarić , J. Alagić , and M. Maksimović . 2014. “Determination of Total Phenolic Content and Antioxidant Activity of Ethanol Extracts From Aloe spp.” Bulletin of the Chemists and Technologists of Bosnia and Herzegovina 42: 5–10.

[fsn371264-bib-0055] Wang, Y. , X. Li , L.‐H. Li , D.‐L. Meng , Z.‐L. Li , and N. Li . 2007. “Two New Thiophenes From *Echinops latifolius* and Their Phototoxic Activities.” Planta Medica 73, no. 7: 696–698. 17564953 10.1055/s-2007-981541

[fsn371264-bib-0056] Yahya, R. , A. M. Al‐Rajhi , S. Z. Alzaid , et al. 2022. “Molecular Docking and Efficacy of *Aloe vera* Gel Based on Chitosan Nanoparticles Against Helicobacter Pylori and Its Antioxidant and Anti‐Inflammatory Activities.” Polymers 14, no. 15: 2994. 35893958 10.3390/polym14152994 PMC9330094

[fsn371264-bib-0057] Yazdi, F. T. , B. A. Behbahani , A. Vasiee , S. A. Mortazavi , and F. T. Yazdi . 2015. “An Investigation on the Effect of Alcoholic and Aqueous Extracts of *Dorema aucheri* (Bilhar) on Some Pathogenic Bacteria In Vitro.” Archives of Advances in Biosciences 6: 1.

[fsn371264-bib-0058] Yebpella, G. , C. Hammuel , H. Adeyemi , A. Magomya , A. Agbaji , and G. Shallangwa . 2011. “Phytochemical Screening and a Comparative Study of Antibacterial Activity of *Aloe vera* Green Rind, Gel and Leaf Pulp Extracts.” International Research Journal of Microbiology 2, no. 10: 382–386.

[fsn371264-bib-0059] Yusoff, I. M. , Z. M. Taher , Z. Rahmat , and L. S. Chua . 2022. “A Review of Ultrasound‐Assisted Extraction for Plant Bioactive Compounds: Phenolics, Flavonoids, Thymols, Saponins and Proteins.” Food Research International 157: 111268. 35761580 10.1016/j.foodres.2022.111268

